# Dengue Vaccine Development and Deployment into Routine Immunization

**DOI:** 10.3390/vaccines13050483

**Published:** 2025-04-29

**Authors:** Annelies Wilder-Smith, Thomas Cherian, Joachim Hombach

**Affiliations:** World Health Organization, CH-1211 Geneva, Switzerland; cheriant@mmglobalhealth.org (T.C.); hombachj@who.int (J.H.)

**Keywords:** dengue, Qdenga, CYD-TDV, TAK-003, Wolbachia, antibody-dependent enhancement, mRNA vaccines, school-based programs

## Abstract

Dengue has emerged as a significant global health threat. Despite decades of research, only two dengue vaccines—CYD-TDV (Dengvaxia) and TAK-003 (Qdenga)—have been licensed to date, with limited implementation. This paper explores and outlines strategies for integrating dengue vaccines into routine immunization programs, particularly in high-burden regions. TAK-003, a tetravalent live-attenuated vaccine, has demonstrated 61% efficacy against virologically confirmed dengue and 84% efficacy against hospitalizations in endemic settings. However, concerns remain about vaccine-enhanced disease, particularly among seronegative individuals exposed to DENV3 and DENV4. WHO recommends targeted introduction in high-transmission settings without pre-vaccination screening, while ongoing post-introduction studies will further clarify long-term safety and efficacy. Effective vaccine rollout requires a multi-pronged approach, including school-based immunization, integration with adolescent health services, and strong community engagement. Decision-making for vaccine introduction should be guided by National Immunization Technical Advisory Groups (NITAGs), local epidemiological data, and cost-effectiveness assessments. While future vaccines, including mRNA and virus-like particle candidates, are under development, optimizing the use of currently available vaccines is crucial to reducing dengue’s public health impact. Given the continued rise in cases, immediate action—combining vaccination with vector control—is essential to prevent further morbidity and mortality.

## 1. Introduction

Dengue has emerged as one of the leading global health threats, with a significant rise in incidence and geographic spread. Despite decades of research, as of early 2025, only two dengue vaccines have been licensed, neither of which has been widely implemented. This paper examines the key challenges in dengue vaccine development and deployment, including scientific, regulatory, logistical, and policy barriers. It also explores strategies to integrate dengue vaccines into the Essential Programme on Immunization (EPI), ensuring broader access and long-term disease control.

Dengue is caused by any of the four dengue virus (DENV) serotypes, belonging to the family of Flaviviridae, and is primarily transmitted by *Aedes aegypti* and *Aedes albopictus* mosquitoes, which are endemic to most tropical and subtropical regions [1]. The co-circulation of different DENV serotypes varies by region, with some areas experiencing simultaneous outbreaks of multiple serotypes [2,3]. However, one serotype usually dominates at any given time.

The clinical spectrum of dengue ranges from asymptomatic, mild, or subclinical illness to more severe and potentially life-threatening forms of the disease. In hyperendemic areas, symptomatic dengue is primarily a disease of younger children. In moderately endemic countries, it affects the full range of children and younger adults. Additionally, in regions with low-to-moderate endemicity, clinical disease is typically also seen among older persons, as reported in ref. [4], with the frequency and types of complications observed reflecting underlying co-morbidities in this group [5]. Diabetes, hypertension, cardiovascular conditions, and sickle cell disease increase the risk of severe dengue. Pregnant women with dengue also face higher risks of adverse outcomes [6].

With proper clinical management, case fatality rates can be reduced to well below 0.1%. However, the absence of reliable prognostic markers for severe dengue progression often leads to the need for hospitalization and close monitoring of many patients [7]. During outbreaks, this places immense pressure on already fragile healthcare systems, resulting in increased case fatality rates.

## 2. Why Is There a Need for a Dengue Vaccine?

Dengue has experienced an increase in the incidence of cases and number of outbreaks, as well as geographic expansion, and is poised to increase further [8,9]. Approximately 4 billion people are living in regions where *Aedes* mosquitoes are endemic resulting in an estimated 400 million dengue infections annually [10]. The main drivers for the proliferation of *Aedes* mosquitoes and increasing dengue incidence are population growth, rural-to-urban migration, high population densities, unplanned urbanization with substandard water and waste management, global warming, and disorganized and inadequately funded mosquito control programs [8]. Outbreaks are increasing in frequency and magnitude. Regions such as Asia and the Americas are most affected, though recent outbreaks in Africa suggest a larger burden in that continent than previously estimated [11]. Dengue incidence is poised to increase further, both in frequency as well as geographic range. Dengue is also increasingly reported in countries not classically endemic for dengue [12,13,14,15,16,17].

Dengue control has traditionally focused on mosquito control, but scaling up these efforts remains both complex and costly [18]. The continued rise in dengue cases suggests that existing strategies have largely fallen short, and while innovative approaches like *Wolbachia* show promise [19], they require time for full implementation and will not be enough to achieve complete dengue control on their own [20]. To effectively reduce the disease burden, dengue vaccines should be integrated into routine immunization through the Essential Programme on Immunization (EPI) alongside vector control measures.

## 3. What Is the Current Status of Dengue Vaccine Development?

Dengue vaccine development has been ongoing for over 50 years, but numerous challenges have made bringing a vaccine to market difficult. One major obstacle has been the lack of an appropriate animal model, along with a limited understanding of the immune correlates needed for both protection and disease enhancement. Controlled human infection models (CHIMs) have been developed; however, they may not provide sufficient answers given that the infecting virus has to be attenuated and, therefore, does not totally mimic real infection. Furthermore, according to the antibody-dependent enhancement hypothesis (ADE), a safety signal would only be seen after an extended period [21]. However, the most significant challenge lies in the complex immunological interactions between the four antigenically distinct dengue serotypes [22,23]. These interactions often lead to imbalanced vaccine performance, which could, in some cases, increase the risk of ADE.

Over the years, various vaccine platforms have been explored, including live-attenuated chimeric recombinant viruses, live-attenuated viruses, inactivated viruses, recombinant proteins, and mRNA-based vaccines. Despite this broad range of approaches, only live-attenuated vaccines have completed Phase 3 trials. Ideally, a tetravalent live-attenuated vaccine would generate strong, independent immune responses to all four serotypes. However, achieving this balance has proven to be a significant challenge.

There are currently two licensed dengue vaccines: CYD-TDV (Dengvaxia, Sanofi) and TAK-003 (Qdenga, Takeda). Both are tetravalent live-attenuated vaccines but differ in the extent of chimerization and the genome backbone. Another tetravalent live-attenuated dengue vaccine (TV003/005) developed at the Laboratory of Infectious Diseases, at the National Institutes of Allergy and Infectious Diseases (NIAID) in the United States, has completed Phase 3 trials in Brazil, but the final data have yet to be released. All three live-attenuated vaccines use chimerization, and these vaccines differ by the extent of chimerization and the backbone used, as well as in efficacy by serotype and safety.

CYD-TDV, a tetravalent live attenuated with a yellow fever 17D backbone, developed by Sanofi Pasteur, known under the trade name of Dengvaxia^®^, was the first dengue vaccine to be licensed. Phase 3 trials revealed a vaccine efficacy that varied by age, serostatus, and serotype [24]. Although this vaccine has a clear overall population-level benefit, in baseline seronegative persons, this vaccine increases the risk of severe dengue over time when exposed [25,26]. For Dengvaxia^®^, the World Health Organization (WHO) therefore recommends a pre-vaccination screening strategy, whereby only dengue-seropositive persons are vaccinated [27]. Because of the cost and programmatic hurdles for pre-screening before vaccination, the use of Dengvaxia^®^ in national immunization programs has been very limited. For commercial reasons, the company has therefore decided not to further manufacture this vaccine, and it will be discontinued.

The advantages of TAK-003 and TV003/005 over CYD-TDV are the inclusion of non-structural proteins of the dengue backbone and reduced numbers of doses needed.

TAK-003 was developed by Takeda and is known under the trade name of Qdenga^®^ [28,29]. TAK-003 consists of an attenuated DENV-2 (DEN2-PDK-53) backbone, whereby three chimeric viruses containing the prM and envelope proteins of DENV-1, -3, and -4 are inserted into the DEN2-PDK-53 backbone. The advantage of TAK-003 to CYD-TDV, therefore, is the presence of more non-structural proteins due to the DENV2 backbone. TAK-003 induces high levels of DENV2-neutralizing antibodies against DENV2, but lower levels of DENV1-, DENV3-, and DENV4-neutralizing antibodies [30]. Various studies and Phase 1–3 trials have been published on TAK-003 (Qdenga) [28,31,32,33,34,35,36,37,38,39,40,41,42,43,44,45,46,47]. Of note, no important safety risks were identified, and TAK-003 was well tolerated irrespective of age, gender, or baseline dengue serostatus in recipients aged 4–60 years [48].

In the pivotal trials conducted in dengue endemic countries, the vaccine efficacy (VE) of TAK-003 over 5 years against virologically confirmed dengue (VCD) was 61% (95% CI 56.0, 65.8), and against dengue-related hospitalizations the vaccine efficacy was 84% (77.8–88.6) [28,37]. TAK-003 demonstrated efficacy against all four serotypes among baseline seropositive subjects and against DENV1 and DENV2 in baseline seronegative subjects. There was no efficacy in baseline seronegative subjects against DENV3 and 4.

Modeling indicates that the population-level benefit of this vaccine will be substantial, with the public health impact highest in settings with high seroprevalence (e.g., high proportion of seropositive persons indicating high burden of disease) and in settings where serotype 2 is circulating [49].

The large pivotal Phase 3 trial involving about 20,000 children was not sufficiently powered to definitively rule out a risk of vaccine-enhanced disease in seronegative persons exposed to DENV3 and DENV4 as the number of cases due to DENV3 and DENV4 was relatively small during the trial period. There were higher rates of symptomatic as well as hospitalized DENV3 cases amongst seronegative vaccinated persons than among seronegative unvaccinated persons, but this excess was small and not statistically significant. WHO acknowledges that vaccine-enhanced disease in a subpopulation of seronegative persons exposed to DENV3 and possibly DENV4 is biologically plausible and cannot be excluded from the results of the Phase 3 trial.

Modeling suggests that at a population level, after 10 years of vaccination, seronegative persons will have a net benefit, even in relation to DENV3 and DENV4 [29]. However, statistical uncertainty remains. What is clear is that TAK-003 will have the greatest public health impact and be the most cost-effective in settings with high dengue transmission intensity.

The available trial data indicate that the benefits of this vaccine significantly outweigh the risks. Like many other vaccines, this vaccine is only partially protective. Breakthrough infections can occur in vaccinated persons, in both seropositive and seronegative persons at the time of vaccination, and such breakthrough infections can be associated with mild to severe disease. On an individual basis, breakthrough disease cannot be distinguished from potential cases of enhanced disease. The risk of enhanced disease can only be determined in population-based studies with a long observation time. These data may not become available for at least five years, pending post-introduction studies.

## 4. How to Use TAK-003 (Qdenga) in Endemic Populations?

The WHO recommends that countries consider introducing the TAK-003 dengue vaccine into routine immunization programs in areas where dengue poses a significant public health burden due to high transmission intensity. Since dengue transmission varies geographically, some countries may opt for a targeted rollout in specific subnational regions with higher transmission rates rather than a nationwide approach.

Seroprevalence is the proportion of seropositive persons in a population and a measure of the force of infection. The higher the force of infection, the higher the seroprevalence at a younger age [50]. Higher seroprevalence at younger ages indicates greater dengue transmission intensity.

To determine whether a region qualifies as a high-transmission setting, countries should assess data on age-specific seroprevalence and/or age-specific dengue hospital admissions. While there is no exact seroprevalence threshold that indicates when vaccination should be introduced, the benefits of the vaccine increase with higher seroprevalence, particularly in individuals who have already been exposed to dengue. As a general guideline, a seroprevalence of more than 60% by the age of 9 years indicates high transmission, while a mean age of peak dengue-related hospitalizations well below 16 years could also serve as an indicator. A map on national and subnational levels of seroprevalence rates was published to guide countries in their decision to introduce TAK-003: https://arbomap.org/ (accessed on 10 February 2025).

In settings with high dengue transmission, pre-vaccination screening to limit vaccination to seropositive individuals is not recommended, as it would significantly reduce the public health impact and increase programmatic costs. Instead, vaccine introduction should be supported by a well-designed communication strategy and strong community engagement to ensure public trust and awareness.

In areas with low-to-moderate dengue transmission, the WHO does not recommend the programmatic use of TAK-003 until more data are available on its efficacy and risk profile in seronegative individuals, particularly for DENV3 and DENV4 [29,51].

## 5. What About the Reported Cases of Anaphylaxis Post-Vaccination?

Whilst during the clinical Phase 3 trials involving more than 20,000 participants in two continents no cases of anaphylaxis were observed, during vaccine roll-out, cases of anaphylaxis and hypersensitivity reactions were reported. In the initial roll out in Brazil, of 380,358 doses of Qdenga administered, 626 AEFI were reported [52]. Of these, 85 were cases of immediate hypersensitivity, with 24 (63.1 cases per million) being anaphylaxis, including three cases of anaphylactic shock. For 42% of these cases, hypersensitivity reactions began within 15 min after vaccination. In view of these hypersensitivity reactions, the Ministry of Health of Brazil published recommendations for intensifying actions for safe vaccination, including healthcare professional training and post-vaccination observation [52].

The WHO’s Global Advisory Committee for Vaccine Safety (GACVS) published in November 2024 that in the vaccine roll-out in Brazil and beyond with more than 2 million doses, between 1 March 2023 and 12 September 2024, 501 hypersensitivity reactions (147.1 per million doses) were reported after vaccination with Qdenga^®^ [53]. Of these, 447 occurred after the first dose (183.1 per million doses), and 24 occurred after the second dose (31.0 per million doses). Of all the reactions, 124 were classified as anaphylaxis (36.4 per million doses), with 117 after the first dose (44.5 per million doses) and 7 after the second dose (9.0 per million doses). In Brazil, immunization outreach activities are being replaced by in-facility vaccination [53]. GACVS states that for children with mild-to-moderate reactions, a supervised second dose can be given [53]. A second dose is contraindicated for those with confirmed anaphylaxis. A research project is under way to investigate risk factors, including possible allergic components of the vaccine and the mechanisms of the reactions [53].

No deaths related to anaphylaxis have been reported to date.

## 6. What Are the Programmatic Considerations for Including TAK-003 (Qdenga) in Routine Immunization?

To effectively roll out TAK-003, identifying high transmission areas is essential. Conducting seroprevalence studies can guide targeted vaccination strategies, ensuring those at highest risk are prioritized while reducing the risk of adverse events in dengue-naïve individuals. Documenting peak incidence of hospitalization is a further aid.

Education and awareness campaigns are vital to improve vaccine acceptance and build public trust. Transparent communication about the benefits and potential risks—especially the possibility of increased disease severity in certain epidemiological contexts—is critical for informed decision-making.

The vaccine is recommended for school-aged children and adolescents (ages 6–16, per WHO guidance). This requires strong platforms for school- and adolescent-based immunization. Co-administering TAK-003 with other routine vaccines, such as HPV and tetanus-diphtheria, can increase coverage and lower operational costs. Vaccination can be organized by school grade, class, or age to ensure broad access and integration into existing school health services. Schools with dedicated health personnel can more easily incorporate vaccination into routine programming. Facilities must also be equipped to manage anaphylaxis, with extended post-vaccination observation periods to ensure rapid response. For school-based programs, portable emergency kits should be at hand, as well as an effective referral system to higher level care, should it be needed.

To achieve high uptake, it is essential to foster public confidence, supportive social norms, and deliver high-quality services. Introduction strategies should be tailored to local contexts, accounting for epidemiological trends, demographic factors, and available resources. This includes prioritizing areas with high transmission, assessing age-specific seroprevalence, and focusing on regions with high dengue-related hospitalization rates.

Community engagement, risk communication, and context-specific messaging should precede rollout. Studies on behavioral and social drivers of vaccine uptake can help design more effective interventions. Efforts should also reinforce vector control and environmental management to support broader dengue prevention.

For out-of-school children and adolescents—such as those who are homeschooled, older than school age, or in areas without school-based programs—non-school-based strategies are essential. These include outreach campaigns, community clinics, and integration with other adolescent health services like counseling and screenings. Utilizing accessible locations and community networks helps overcome logistical barriers and expand coverage.

Outreach and national vaccination campaigns are particularly important in underserved areas. Mobile health teams can deliver vaccines directly to communities. Strategies like holding two national immunization days three months apart, followed by annual boosters, can quickly increase uptake.

Vaccination through clinics and healthcare facilities provides a safe, monitored environment. Hosting dedicated vaccination days, paired with incentives like shorter wait times and educational sessions, can boost participation. Schools can also host on-site vaccination events, making the process more accessible and less intimidating.

To maximize coverage—especially in rural and underserved areas—a mix of school-based, clinic-based, and outreach approaches is often necessary.

Effective implementation depends on coordinated involvement from all stakeholders. **In brief, government officials** need data-driven evidence to justify national adoption. **Health and education authorities** must align vaccine rollout with broader public health goals. **Schools**, supported by trained staff and adequate resources, play a key role in implementation. **Health workers**, as frontline communicators, must be equipped to address community concerns. **Community organizations**, including professional, religious, and cultural groups, can help tailor messaging to diverse audiences. **Politicians and the media** shape public perception and must be informed advocates for the vaccine.

Tailored engagement with each stakeholder group is critical for successful vaccine uptake and long-term public health impact.

## 7. What Are the Key Steps in Deciding Whether a Dengue Vaccine Should Be Introduced into a Routine Immunization Program?

A structured and transparent process is essential for making decisions about introducing a dengue vaccine. Governments should be guided by recommendations from the National Immunization Technical Advisory Group (NITAG), input from relevant stakeholders, and an assessment of public health needs and feasibility.

NITAG—or an equivalent advisory body—should conduct an independent, evidence-based review of the vaccine’s suitability. This ensures credibility, minimizes external influence, and supports funding decisions. The process, led by the National Immunization Program (NIP), should align with national decision-making frameworks and include the evaluation of the local dengue burden and transmission intensity.

Following established steps (such as those outlined in Box 1) enables a well-coordinated, evidence-informed vaccine introduction that strengthens immunization programs and improves health outcomes.

Box 1 **Key Steps in the Decision-Making Process.**

- **Engage Key Stakeholders**
  ○ Involve representatives from **public health, government agencies, and advocacy groups**.
  ○ High-level advocates, such as government officials or influential figures, can help **drive discussions and secure support**.
- **Consult NITAG**
  ○ Seek **expert review** of the dengue vaccine’s evidence and recommendations.
  ○ Engage **regional dengue specialists** if additional expertise is needed.
- **Establish a Technical Working Group (TWG)**
  ○ Form a **multi-sectoral team** of experts in immunization, infectious diseases, and public health to **provide technical guidance and coordination**.
- **Prioritize High-Transmission Regions**
  ○ Identify **areas with the highest dengue burden** for targeted rollout.
  ○ Develop a **phased introduction plan** based on subnational needs to **optimize resource allocation**.
- **Integrate into National Strategies**
  ○ Upon approval, incorporate the vaccine into the **National Immunization Strategy** and **Primary Health Care Strategy**.
  ○ Define **funding, implementation, and monitoring** frameworks.
- **Develop Vaccination Plans**
  ○ Create **locally tailored plans** prioritizing **school-age children and adolescents**.
  ○ Design **micro plans** within existing immunization structures.
- **Leverage Existing Vaccination Platforms**
  ○ Utilize **school-based and adolescent vaccination programs**.
  ○ Consider **co-administering with other vaccines** (e.g., **HPV**, **tetanus**) and integrating with **other health initiatives** (e.g., **WASH programs**) to enhance coverage.
- **Establish Governance Structures**
  ○ Set up **national, subnational, and district-level committees** to oversee vaccine **procurement, distribution, safety, and surveillance**.
- **Set Subnational Targets**
  ○ Define clear **coverage goals**, aiming for **at least 90% vaccination** in target populations.
  ○ Integrate with **school-based health programs** and include **catch-up vaccinations** for broader age groups.

## 8. What Is the Modeled Impact of TAK-003 (QDenga) in Endemic Settings?

A mathematical model developed at Imperial College (London) predicted that TAK-003 would provide high protection against virologically confirmed dengue (VCD) and dengue hospitalizations caused by DENV2 and moderate protection against VCD and hospitalization caused by DENV1 for both seropositive and seronegative children. Simulating 80% routine vaccination coverage at age 6–12 years, the model predicts positive impacts of routine vaccination with TAK-003 at the population level, with clearly larger impacts in higher transmission settings [29]. Overall, the impact is modest with an average proportion of all cases averted not exceeding 15% and 20%, respectively, for VCD and hospitalization [29,49]. Assuming moderate protection against infection gives a higher population impact than assuming no protection, especially in lower transmission settings. Furthermore, a positive average individual-level benefit of vaccination in all strata of the population can be expected, including for baseline seronegative individuals and in cases of primary breakthrough infections. However, the analysis suggests that the 95% range of possible outcomes includes a small probability of negative outcomes associated with DENV3 and DENV4 primary breakthrough infections. Pre-vaccination serological screening could largely, although not completely, remove any potential risk to seronegative individuals; however, such screening is estimated to halve the population-level impact of vaccination, impose substantially increased programmatic costs, and hamper vaccine uptake. Clearly, with the roll-out of vaccines, empiric data need to be obtained to assess the risk of severe dengue in seronegative persons exposed to DENV3 and 4.

## 9. What About the Use of Qdenga in Travelers?

Travelers are at risk of dengue and contribute to the global spread of DENV [14,15,54,55,56,57]. However, the WHO recommendations focus on how best to use Qdenga in endemic populations rather than travelers [58]. No benefit–risk assessments have been conducted for the use of dengue vaccines in travelers. Nevertheless, the WHO acknowledges its benefit in seropositive travelers visiting highly dengue endemic areas, whilst the benefit in seronegative travelers is lower [50]. Most travelers are seronegative, hence the overall effectiveness of Qdenga in travelers is clearly lower compared to endemic populations with high seropositivity rates. Travelers must be informed that Qdenga has no efficacy against serotypes 3 and 4 if they are seronegative, and a safety signal in such persons has not been proven or disproven. First experiences in the use of Qdenga in travelers have been published [59].

## 10. Which Other Dengue Vaccines Are in the Pipeline?

The Laboratory of Infectious Diseases at the National Institutes of Health (NIAID), United States, has developed five single-dose live-attenuated tetravalent DENV vaccines, of which TV003 and TV005 are the most promising candidates based on the most optimal immunogenicity and safety profile. A relatively balanced immune response to all four serotypes has been documented [60]. Both TV003 and TV005 have undergone extensive Phase 1 and 2 testing, including human challenge studies [61], and the Instituto Butantan is completing a Phase 3 trial using TV003 in Brazil. TV003 and TV005 share the same four monovalent components; the differences are in the dosing for serotype 2. TV005 has a 10-fold higher dose at 10^4^ PFU to potentially overcome the over-attenuated serotype 2 component [61,62]. NIH has licensed out the vaccines to different manufacturers.

Several developers have secured licensure for this vaccine, with Merck having the largest global share. Instituto Butantan owns the licensure for distribution in Brazil. Butantan led the Phase 3 efficacy trial in Brazil in 16 sites, involving 16,235 volunteers aged 2 to 59 years using a single dose schedule. The two-year results have to date only been published in a press release. Over the initial two years, the vaccine was 79.6% effective in preventing dengue (serotypes 1 and 2) [63]. Efficacy in seropositive vs. seronegative persons was 89.2% and 73.5%, respectively. No confidence intervals were provided. The efficacy could only be assessed for serotypes 1 and 2, as serotypes 3 and 4 were not circulating during those initial two years. Efficacy against DENV-1 was higher with 89.5% versus 69.6% for DENV-2. By mid 2025, the results of the 5-year trial observation time are expected to become publicly available. Submission of the regulatory dossier to the European Medicine Agency has already taken place.

Recent advancements in dengue vaccine development have explored several additional innovative platforms beyond traditional live-attenuated vaccines. These include mRNA vaccines, which may offer a scalable and flexible approach to targeting multiple dengue virus serotypes [64]. Virus-like particle (VLP) vaccines mimic the structure of viruses without containing viral genetic material, eliciting strong immune responses while being non-infectious. A study published in 2024 describes the development of a tetravalent dengue VLP vaccine that induced robust immune responses against all four serotypes in animal models, maintaining efficacy for over a year [65]. Subunit vaccines use specific protein fragments of the dengue virus to stimulate immunity. A bioinformatics-driven approach has been proposed to design multi-epitope subunit vaccines targeting conserved regions of dengue virus proteins, aiming to induce comprehensive immune responses [66].

These innovative platforms aim to overcome the imbalanced performance of live-attenuated vaccines by eliciting balanced immunity against all four dengue virus serotypes. However, none of these platforms have moved beyond animal or Phase 1 studies. Ongoing research and clinical trials are crucial to evaluate the safety, efficacy, and scalability of these novel vaccine candidates. Given the enormous costs and complexities of running large Phase 3 trials, there is an urgent need to identify correlates of protection and, perhaps even more importantly in the context of antibody dependent enhance, correlates of enhanced disease [67].

As yet, no single vaccine can fully prevent and eliminate dengue infections. Hence, dengue vaccines will need to be used in conjunction with vector control [9].

Although Zika virus infections are currently rare, the risk of re-emergence with its catastrophic consequences of congenital Zika disease would justify developing a combination vaccine protecting both against dengue and Zika; however, several challenges persist which make such a vaccine less likely, as recently reviewed [68].

## 11. Conclusions

There is no perfect dengue vaccine, just as there is no vaccine with 100% efficacy for any other disease. The focus should be on optimizing the use of currently licensed dengue vaccines to maximize public health benefits while minimizing risks. Waiting for a perfect dengue vaccine will only result in more dengue morbidity and preventable deaths. Instead, we must act now, using available vaccines—despite their limitations—alongside effective vector control measures to reduce the burden of dengue and save lives. A well-planned vaccine roll-out combined with transparent communication, epidemiological monitoring, surveillance (serotype and genomic surveillance), and post-introduction studies to determine its safety in seronegative persons is needed.
